# Survival from adult leukaemia in England and Wales up to 2001

**DOI:** 10.1038/sj.bjc.6604610

**Published:** 2008-09-23

**Authors:** D W Milligan

**Affiliations:** 1 Department of Haematology, Birmingham Heartlands Hospital, Birmingham B9 5SS, UK

Leukaemia comprises four main groups of conditions: acute myeloid leukaemia (AML), acute lymphoblastic leukaemia (ALL), chronic lymphocytic leukaemia (CLL) and chronic myeloid leukaemia (CML). Acute lymphoblastic leukaemia is mainly seen in children and AML is most commonly seen in adults and half of all are aged more than 60 years. Chronic lymphocytic leukaemia is a condition of the elderly and CML is seen in most age groups from older children onwards.

## Clinical presentation

The presenting features of leukaemia depend very much on the type. Acute leukaemias tend to have quite a sudden onset, usually over a few weeks, with symptoms of bone marrow failure resulting in infection, bruising and tiredness or dyspnoea on account of anaemia. Some patients, particularly children, may have bone pain as a presenting feature. There are no characteristic symptoms that separate the clinical features of AML and ALL. Chronic lymphocytic leukaemia is now diagnosed in 80% of patients as a chance finding of lymphocytosis in a patient having a blood test for some other condition. Symptomatic patients may present with lymphadenopathy or splenomegaly, and may have symptoms of anaemia or recurrent infections because of low immunoglobulin levels. Chronic myeloid leukaemia is associated with a very high white cell count and significant splenomegaly. The condition can develop insidiously and usually results in symptoms of tiredness owing to anaemia, weight loss or night sweats, and abdominal bloating or discomfort because of splenomegaly.

## Diagnosis

The first step in the diagnosis is usually for the primary care physician to arrange a blood count. Morphological examination of this by an expert is often enough to give reasonable certainty of the diagnosis; however, in the majority of instances, a bone marrow aspirate and biopsy will be required to confirm the diagnosis. Exceptions to this may be in the elderly where active treatment is not offered and the peripheral white cell count is unequivocal. Immunophenotyping of leukaemia cells is key to confirming the diagnosis in acute leukaemias and CLL. Increasingly, cytogenetic analysis, by conventional banding, FISH and PCR for informative translocations and deletions, allows subclassification of leukaemia and prognostic stratification. This is now used to inform clinical trial design where patients falling into poor prognostic categories may be offered different treatments. There are two areas where cytogenetic evaluation is critical. These are acute promyelocytic leukaemia associated with the *PML-RARA* fusion gene, *t*(15;17), and CML characterised by the Philadelphia chromosome (*t*(9;22)). It is probable that in the future, additional information about diagnosis and prognosis will be provided by gene profiling and proteomics, but these are not presently part of routine diagnostic practice.

## Treatment

### Acute myeloid leukaemia

Acute myeloid leukaemia is principally a disease of the elderly. For fitter patients, induction therapy is usually a combination of anthracycline with cytarabine. Depending on age, between 60 and 80% of patients will achieve complete remission. Consolidation treatment of a further two to four additional cycles is usually given. For younger patients, an overall survival (OS) of between 40 and 50% can be attained, but the results are not as good for those aged more than 60 years. The reasons for this are the greater frequency of adverse cytogenetic abnormality and increased P-glycoprotein activity together with increasing comorbidities. It is likely that further increase in dose intensity will not be possible because of toxicity and newer strategies are needed. These may include targeted therapy using FLT3 inhibitors and HIDAC inhibition. Early results of targeted immunotherapy with gemtuzumab ozogamycin are also encouraging. Trials of stem cell autografting have not demonstrated superiority. Allografting has a powerful antileukaemic effect, but this is counterbalanced by high toxicity resulting in a relatively small OS gain. Conventional myeloablative allografting is applicable to only a very small proportion of the overall AML population because of age and donor availability. Reduced intensity allografts may be safer and more easily deliverable to an older population but their efficacy has yet to be established. In older patients with comorbidity treatment outcomes have not improved. Low-dose cytarabine increases OS compared with supportive care, but the results remain very poor. New ideas are needed for this group, as it is apparent that conventional chemotherapy adds little except to a small cohort with good-risk cytogenetics.

### Chronic lymphocytic leukaemia

Overall, 80% of patients with CLL will be diagnosed with a chance blood test. Of these only about one-quarter will require treatment and the majority will die of a cause other than CLL. When treatment is needed, UK practice has been to use chlorambucil; however, a recent trial indicated that better and more durable disease responses can be achieved using fludarabine and cyclophosphamide, in combination. NICE currently does not support the use of fludarabine monotherapy as first-line treatment in CLL. Newer treatments include antibody treatment with rituximab and alemtuzumab and it remains to be seen how the advent of these agents will influence the natural history of the disease. Currently, CLL remains incurable without a stem cell allograft and allografting is not widely applicable to this population.

### Chronic myeloid leukaemia

The standard of care for the duration of this study was *α*-interferon. Patients unable to tolerate this were treated with hydroxycarbamide. Patients treated with interferon had a median duration of survival of approximately 5 years. Younger patients with a donor were offered a stem cell transplant resulting in an OS for this cohort of between 40 and 70%, depending on age. The advent of the tyrosine kinase (TK) inhibitor, imatinib, has now changed the landscape in CML. The majority of patients will now achieve a cytogenetic complete response with some patients having disease undetectable by sensitive PCR tests. For patients treated since 2000, median survival has not yet been reached and 90% of patients can now expect to live 5 years. The newer TK inhibitors may overcome TK mutations and may further improve the outlook.

## Commentary

The principal difficulty that I have as a clinician with the epidemiological data presented by Rachet *et al* in this issue, pp S116–S118 is the grouping of all types of leukaemia together. Even within AML there is considerable heterogeneity and we can subclassify patients according to cytogenetic and other risk factors. Sequential trial data from the UK MRC trials (now NCRI) has demonstrated a continuous improvement in outcomes, particularly in younger patients. This is in part because of better supportive care and the ability to deliver more toxic regimens. Some cytogenetic subgroups are doing particularly well and the introduction of differentiation treatment with all-trans retinoic acid or arsenic trioxide in acute promyelocytic leukaemia has made a very substantial impact in this cohort. In CML the TK inhibitors are almost certainly making a substantial impact on survival but this is not yet being seen in population based studies, partly because the data is too immature and partly because as a population of all patients with leukaemia the numbers are small. However, these real gains are lost in the noise generated by the lack of precision of the data. For this information to become really meaningful we will need much better classification of leukaemia at registration.

